# Assessing the Correlation Between Patient-Specific Characteristics and Braden Pressure Injury Risk Score at a Suburban Tertiary Hospital in Nigeria

**DOI:** 10.7759/cureus.39373

**Published:** 2023-05-23

**Authors:** Adedayo I Salawu, Tope M Ipinnimo, Tolulope A Bamidele, Olakunle F Babalola, Cecilia K Okunlola, Maryam O Adeleye, Precious E Nkereuwem

**Affiliations:** 1 Department of Surgery, Federal Teaching Hospital, Ido-Ekiti, NGA; 2 Department of Surgery, Afe Babalola University, Ado-Ekiti, NGA; 3 Department of Community Medicine, Federal Teaching Hospital, Ido-Ekiti, NGA; 4 Department of Medicine, Federal Teaching Hospital, Ido-Ekiti, NGA; 5 Department of Medicine, Afe Babalola University, Ado-Ekiti, NGA

**Keywords:** risk, pressure ulcer, pressure injury, nigeria, braden score

## Abstract

Background and objective

Pressure injury (PI) is a frequent complication of hospital admissions, and it increases healthcare costs, morbidity, and mortality. This study aimed to use the Braden scale to assess the PI risk among hospitalized patients without PI and determine its association with patient-specific factors.

Methods

A cross-sectional study was conducted at a suburban tertiary hospital involving a total of 211 hospitalized patients recruited during the study period (October 2022). Patients were assessed using the Braden scale and their sociodemographic data were also recorded. Data analysis was performed using IBM SPSS Statistics version 26.0 (IBM Corp., Armonk, NY).

Results

The mean age of the patients was 41.8 ±23.0 years and 54.0% of the patients were females. The average Braden score of the patients was 19.01 ±3.42, and more than half (58.3%) of the patients showed no risk while only 0.9% of the patients had a severe risk of PI. There was a statistically significant association between PI risk and patients’ age (r=-0.353, R=12.5%, p<0.001), pre-admission occupation (F=10.290, p<0.001) as well as the ward of admission (F=11.915, p<0.001). However, gender and social support were not significantly associated.

Conclusion

The age, pre-admission occupation, and ward of admission of patients were found to be significantly associated with the risk of developing PI. It is recommended that more resources be channeled toward preventing PI among high-risk patients in resource-limited settings.

## Introduction

Pressure injury (PI) is a clinical condition also known as bedsore, decubitus ulcer, pressure sore, and pressure ulcer. It is defined as localized damage to the skin and underlying soft tissue usually over a bony prominence or related to medical or other devices [1]. PI is a common problem associated with increased morbidity, mortality, and cost of care in patients. In 2016, the United States (US) hospital-acquired PI cost was estimated to exceed $26.8 billion [2,3]. The overall incidence of pressure ulceration during admission ranged from 2.7 to 29% in the US [4,5]. In Nigeria, Onigbinde et al. found an overall incidence rate of 13.84% among all inpatients, half of whom had spinal cord injuries [6].

PI is now recognized as having multi-factorial etiology including friction, shear force, moisture, nutrition, and infection. It was once assumed to originate from direct pressure to soft tissue exceeding the pressure found in the blood vessels supplying the affected area. In order to appropriately stage wounds, identify and treat wound infections, limit risk factors for recurrence, as well as maximize wound healing capacity, there is a need for a multidisciplinary evaluation and treatment of PI. Prevention and treatment of pressure sores should focus on correcting risk factors, and reserving operative intervention until the patient is optimized [7,8].

If practical interventions, like skin examination, risk assessment, bed and chair support surfaces, repositioning, and mobilization as well as nutritional support, are put into practice, the majority of PI incidences can be avoided [9]. In order to identify high-risk patients and institute effective interventions for the prevention of PIs, it is important to employ a reliable risk assessment tool [10]. Many risk assessment tools and measures have been established to estimate the risk of PIs. These include the Norton scale, Waterlow scale, and Braden scale. When compared to other scales, the Braden scale is the most widely used one due to its simplicity of use and inclusion of more risk factors such as moisture and sensory perception. However, it has been applied in several different populations and clinical contexts, with a range of re-verification outcomes [11-15].

The Braden score was developed in 1984 by Braden and Bergstrom. The scale evaluates six elements that contribute to the risk of PI development, which include sensory perception, mobility (ability to change own position), nutrition, moisture, friction or shear, and activity. Each of these elements is scored between 1 and 4. The lower the score, the greater the risk of PI. A score of 19 and above means no risk, between 15 and 18 implies low risk, 13 and 14 implies moderate risk, 10 and 12 implies high risk, while a score of 9 and below signifies severe risk [16]. Several studies have attempted to assess the reliability of Braden scores in dark-skinned individuals such as African-Americans and Hispanics [17,18]. By using a cut-off score of 18, Courtney et al., in a US study, found a high positive predictive value (PPV), sensitivity, and specificity among patients above 75 years of age [17,18].

This study aims to use the Braden scale in assessing the risk of developing PI and evaluate the associated factors among African patients without PI. We believe that the data derived from this study will help hospital managers to plan the allocation of scarce human and material resources better for the prevention of PIs in resource-poor settings.

## Materials and methods

This was a cross-sectional study performed at a 300-bed multi-specialist, referral tertiary hospital that serves several local government areas and receives referrals from other peripheral health centers and hospitals in the Ekiti State, Southwest Nigeria. The hospital has various specialty wards including Internal Medicine, Surgery, Obstetrics, ICU, and Orthopedics among others.

All newly admitted patients into the various wards (Medical, Surgical, Obstetrics and Gynecology, Pediatrics, Orthopedics, and ICU) between the 1st and 31st of October, 2022 were included in the study. The exclusion criteria were as follows: patients who had pre-existing PI at the time of data collection as well as patients in the Accident and Emergency Ward (this is because the maximum duration of admission in the Accident and Emergency Ward is 48 hours). The hospitalized patients were assessed using the Braden scale [16] and their sociodemographics, as well as data on social support (the presence of an individual who is not a healthcare worker and who may be a family or non-family member dedicated to the care of the patient for at least 12 hours in a day), were recorded. The Braden scale is divided into six subscales: sensory perception, skin moisture, friction and shear, nutritional status, activity, and mobility [16]. The data collection was carried out by three doctors and all data were recorded within the first week of admission at the first encounter with the patient. The data collectors (doctors) worked individually after receiving prior training from the principal investigator and no patient was visited more than once. The patients were recruited consecutively and a total of 211 patients were recruited during the study period.

The data were input and refined using the Microsoft Excel spreadsheet and then imported into IBM SPSS Statistics for Windows software version 26.0 (IBM Corp., Armonk, NY) for analysis. Descriptive statistics such as frequency tables and percentages were used to present the sociodemographics and other patient variables. The Braden score was employed based on the description by Bergstrom et al. [16]. Mean and standard deviation (SD) were used to summarize the age of patients as well as the Braden PI risk score. Furthermore, patients were categorized into different classes of PI risk based on the Braden score (≥19: no risk; 15-18: low risk; 13-14: moderate risk; 10-12: high risk; and ≤9: severe risk) [19]. The test of significant association was done using the student's-t-test to compare two means and a one-way analysis of variance to compare more than two means. The association between age and Braden PI risk score was determined by the Pearson correlation coefficient. A p-value <0.05 was considered statistically significant.

Ethical clearance (protocol number: ERC/2022/08/30/884A) was requested and received from the Human Research and Ethics Committee of the Federal Teaching Hospital, Ido-Ekiti. Informed consent was obtained from the patients before including them in the study. The study was conducted in line with the tenets of the Declaration of Helsinki for studies involving human subjects, including research on identifiable human material and data.

## Results

A total of 211 patients were admitted to various wards during the study period. All patients were black Africans. As shown in Table 1, the mean age of the cases was 41.8 ±23.0 years; 46.0% of the patients were males and 54.0% were females. Over one-third of the patients were working in the informal sector of the economy, including clergymen, farmers, and retail traders prior to their admission, while 12.3% and 12.8% were unemployed and retired respectively. One-fifth (20%) of the patients were working in the formal sector, such as clerical officers, professionals, and civil servants prior to needing hospitalization. In terms of ward distribution, the Medical Ward had the highest number of patients with 31.8% of the patients admitted for medical illnesses such as diabetes mellitus and cardiovascular disorders. This was followed by the Surgical Ward with 24.2%; this group of patients tended to have malignancies and acute abdominal and urological disorders. The obstetrics patients constituted 16.6% of the cases, the majority of whom were patients in the immediate puerperal period. Other wards included Pediatrics (15.1%), Orthopedics (10.4%), and ICU (1.9%). The majority (98.6%) of the patients had social support.

**Table 1:** Sociodemographic and other characteristics of the patients SD: standard deviation

Variable	Frequency (N=211)	Percentage (%)
Age, years, mean ±SD	41.75 ±22.95	
Sex		
Male	97	46.0
Female	114	54.0
Occupation		
Informal	79	37.4
Formal	43	20.4
Retired	27	12.8
Unemployed	26	12.3
Others	36	17.1
Ward		
Medical	67	31.8
Surgical	51	24.2
Obstetrics and Gynecology	35	16.6
Pediatrics	32	15.1
Orthopedics	22	10.4
Intensive Care Unit	4	1.9
Social Support		
Yes	208	98.6
No	3	1.4

The average total score of the patients in the study was 19.01 ±3.42, with an average moisture score of 3.82 ±0.454 contributing the most, while the friction and shear score was 2.57 ±0.654, contributing the least to the total score (Table 2).

**Table 2:** Average Braden pressure injury score of the patients SD: standard deviation

Braden Pressure Injury Scale	Score, mean ±SD
Sensory Perception	3.79 ±0.514
Moisture	3.82 ±0.454
Activity	2.43 ±1.266
Mobility	3.28 ±0.796
Nutrition	3.17 ±0.730
Friction and Shear	2.57 ±0.654
Total	19.01 ±3.42

More than half (58.3%) of the patients reviewed had no risk of PI while 31.3% had mild risk, 7.1% had moderate risk, 2.4% had high risk, and only 0.9% of the patients had a severe risk for PI (Table 3).

**Table 3:** Pressure injury risk of the patients

Pressure Injury Risk	Braden Score	Frequency (N=211)	Percentage (%)
Severe Risk	<9	2	0.9
High Risk	10–12	5	2.4
Moderate Risk	13–14	15	7.1
Mild Risk	15–18	66	31.3
No Risk	19–23	123	58.3

As shown in Table 4, patient factors such as age, pre-admission occupation, and the ward of admission were associated with the Braden score of the subjects in a statistically significant manner. On the other hand, patient factors such as sex and access to social support were not significantly associated with the Braden score. Age was weakly inversely related to the PI risk and about one-tenth of the variability of this risk was accounted for by the age of the patients (r=-0.353, R=12.5%, p<0.001). The PI risk of the retired patients was higher than those of other occupational categories (F=10.290, p<0.001) while patients admitted to the ICU had the highest risk (F=11.915, p<0.001).

**Table 4:** Association between patient characteristics and pressure injury risk ^r^Pearson correlation coefficient. ^T^Student's t-test. ^F^Analysis of variance SD: standard deviation

Variable	Braden Pressure Injury Score, mean ±SD	Test	P-value
Age		-0.353^r^	<0.001
Sex		-1.493^T^	<0.137
Male	18.63 ±3.444		
Female	19.33 ±3.383		
Occupation		10.290^F^	<0.001
Informal	18.65 ±3.301		
Formal	19.91 ±3.611		
Retired	16.15 ±3.195		
Unemployed	21.35 ±2.038		
Others	19.19 ±2.837		
Ward		11.915^F^	<0.001
Medical	18.94 ±3.700		
Surgical	17.63 ±3.053		
Obstetrics and Gynecology	21.97 ±1.654		
Pediatrics	19.63 ±2.324		
Orthopedics	17.73 ±2.585		
Intensive Care Unit	14.00 ±6.683		
Social Support		-1.990^T^	0.177
Yes	18.97 ±3.423		
No	21.67 ±2.309		

## Discussion

This study correlated the Braden score of newly admitted patients with patient-specific factors such as age, sex, social support, occupational category, and ward of admission of the patients. The wards patients were admitted to were expected to serve as a surrogate assessment of the nature of the clinical condition they were admitted for, as mentioned earlier.

In this study, pertinent findings showed that the average age of patients admitted was 41.8 ±23.0 years and that the age was negatively correlated with the Braden score; this implies that the older the patient's age, the higher the risk of developing PI. This is in keeping with the findings of Chung et al. [15]. In addition, the study by Chung et al. also found a positive correlation between the depth of the ulcer and the age of the patient [15]. The finding of a negative correlation between age and Braden score is not surprising as one would expect older patients to have more debilitating disease conditions due to the aging process. Pre-admission occupational category of the patients also showed retirees to have a higher risk of PIs while the unemployed had no risk of PI. This finding may be explained by the fact that the retirees with a higher risk of PI are more likely to be elderly as the retirement age in Nigeria is 60 years and the unemployed are usually youths.

In terms of the wards of admission, it was found that patients in the ICU had the highest risk of PIs compared with those admitted in other wards such as Obstetrics and Pediatric Wards. Patients in the ICU are typically very ill while, at the other end of the spectrum, most obstetrics patients are relatively healthy and typically do not stay long in the hospital, especially after a normal vaginal delivery. Surgical cases vary from patients having surgeries such as mastectomy to those with intestinal perforations for trauma. This heterogeneous nature of these patients may account for the mild risk score recorded among them. Orthopedic patients are relatively fit but tend to have their mobility limited by the presence of implants and open wounds that may require dressings. These variations in the PI risks are in keeping with previous studies that have shown wide variations in the incidence of PI in different settings [20,21].

The male-to-female ratio of patients included in the study was 1:1.2; no statistically significant difference was noted in their total Braden score, which was expected as both categories of patients are exposed to the same overall clinical and social factors prevalent in the study environment.

Social support is defined as the presence of a caregiver that is not a healthcare worker for at least 12 hours a day, and who may or may not be a financial provider to the cost of care of the patient. In many parts of Nigeria, there is a profound shortage of skilled manpower in the health sector, compounded by the limited coverage of health insurance [22-24]. Social support provides an informal but critical aspect of patient care in our environment and may indeed determine the outcome of care [25,26]. The majority of the patients had some form of social support from the family but the social support did not show any association with the Braden score. This is a shortcoming of the Braden score noted among black African patients in this study because the scoring system does not take into account this important sociodemographic factor in determining the risk of PI during patient care.

The total mean score for patients in this study was 19.01 ±3.42 and more than half (58.3%) of the patients who were recruited did not have a risk of PI. This average score reflects the broad categories of patients recruited into the study; for instance, patients from the Pediatric and Obstetric Wards were included. When the individual components of the Braden scale were analyzed, the findings were similar in ranking order to that of Onigbinde et al., whose study was conducted in Ile-Ife, southwestern Nigeria [27]. Their lowest score was in activity (1.98 ±0.97) while our study also had the lowest score in activity (2.43 ±1.266); the friction and shear score in their study was 2.18 ±0.68 compared to 2.57 ±0.654 in the current study. Third in the ranking was in nutrition in which their score was 2.74 ±0.91 while 3.17 ±0.730 was recorded in the present study. The score in mobility was 3.04 ±0.85, whereas in the current study, it was 3.28 ±0.796, and the sensory score was also higher in our study compared to the Ile-Ife study. The overall score in the Ile-Ife study was 16.93 ±3.44, which placed their patients in the category of “at risk” for PI. Even though the geographical location of the two studies is comparable, the Ile-Ife study was a multi-center study that included both patients with and without PI, unlike our study.

The major limitation of this study is that it was a single-center study, and a relatively small sample size was used. Larger multi-center studies and systematic reviews should be conducted to gain more clarification on the findings noted.

## Conclusions

Based on our findings, older age, occupation, and type of illness/ward of admission a patient is assigned to contribute significantly to the patient’s risk of developing PI. On the contrary, the sex of the patient was not a significant factor. It is recommended that more resources be channeled toward preventing PI in older patients and patients in ICUs at the time of admission as they have a higher risk compared to others.
